# Exploring theoretical mechanisms of community-engaged research: a multilevel cross-sectional national study of structural and relational practices in community-academic partnerships

**DOI:** 10.1186/s12939-022-01663-y

**Published:** 2022-05-02

**Authors:** John G. Oetzel, Blake Boursaw, Maya Magarati, Elizabeth Dickson, Shannon Sanchez-Youngman, Leo Morales, Sarah Kastelic, Milton “Mickey” Eder, Nina Wallerstein

**Affiliations:** 1 University of Waikato, Waikato Management School, Hamilton, New Zealand; 2 University of New Mexico, College of Nursing, Albuquerque, USA; 3 Department of Psychiatric and Behavioral Sciences, University of Washington, Seven Directions: A Center for Indigneous Public Health, Center for the Study of Health and Risk Behaviors, School of Medicine, Seattle, USA; 4 University of New Mexico, Center for Participatory Research, Albuquerque, USA; 5 University of Washington, School of Medicine, Seattle, USA; 6 National Indian Child Welfare Association, Portland, USA; 7 Department of Family Medicine and Community Health, University of Minnesota, Minneapolis, USA

**Keywords:** Community-based participatory research (CBPR), Community-engaged research, CBPR conceptual model, Participatory health research, Collective empowerment

## Abstract

**Background:**

Community-Based Participatory Research (CBPR) is often used to address health inequities due to structural racism. However, much of the existing literature emphasizes relationships and synergy rather than structural components of CBPR. This study introduces and tests new theoretical mechanisms of the CBPR Conceptual Model to address this limitation.

**Methods:**

Three-stage online cross-sectional survey administered from 2016 to 2018 with 165 community-engaged research projects identified through federal databases or training grants. Participants (*N* = 453) were principal investigators and project team members (both academic and community partners) who provided project-level details and perceived contexts, processes, and outcomes. Data were analyzed through structural equation modeling and fuzzy-set qualitative comparison analysis.

**Results:**

Commitment to Collective Empowerment was a key mediating variable between context and intervention activities. Synergy and Community Engagement in Research Actions were mediating variables between context/partnership process and outcomes. Collective Empowerment was most strongly aligned with Synergy, while higher levels of Structural Governance and lower levels of Relationships were most consistent with higher Community Engagement in Research Actions.

**Conclusions:**

The CBPR Conceptual Model identifies key theoretical mechanisms for explaining health equity and health outcomes in community-academic partnerships. The scholarly literature’s preoccupation with synergy and relationships overlooks two promising practices—Structural Governance and Collective Empowerment—that interact from contexts through mechanisms to influence outcomes. These results also expand expectations beyond a “one size fits all” for reliably producing positive outcomes.

**Supplementary Information:**

The online version contains supplementary material available at 10.1186/s12939-022-01663-y.

## Introduction

Public health is at a crossroads with the dual pandemics of COVID and structural racism producing alarming health inequities [1]. In response, community engagement has emerged as a key global health strategy, with its power to invoke community resilience and actions [2–6]. A substantial body of literature recognizes this strategy, with evidence of impressive impacts of community-engaged research (CEnR) on health equity and health outcomes [5, 7, 8]. It is because of its deep value base of shared power through principled engagement, rather than utilitarian reasons [2, 9], that community-based participatory research (CBPR) is the most commonly-used form of community engagement, accounting for 62% in a systematic review of CEnR studies addressing health inequities [8]. With this promise, there is an urgent need to understand the key partnership practices that make a difference [2, 10].

The primary research approach for CEnR has been to examine relationships, trust, and synergy [10–12]. In addition to relational practices, our large-scale study of community-academic partnerships in the U.S. provides evidence of the importance of partnership structural practices. By providing a mixed-methods analysis of 165 U.S. research partnerships, this study showcases the interaction of both structural and relational pathways, with specific empowerment practices, that together are needed to motivate societal transformations that address the dual pandemics and health inequities facing global public health.

Given the recognized importance of CEnR, researchers and practitioners have sought models and measures to identify promising practices and key approaches to improve practice and health outcomes [13–15]. Also needed to improve collaborative research and practice is better understanding of theoretical pathways, moving from a specific community context through mechanisms to health outcomes and inequities [10, 16]. We start by reviewing three models to introduce key theoretical pathways: a) CBPR Conceptual Model; b) Partnership Evaluation Study logic model and c) a middle-range theory model. Table 1 provides an overview of the model paths organized using the context- mechanism-outcome framework [10, 16]. We then discuss a revision of the CBPR Conceptual Model that is tested in this study.

**Table 1:**
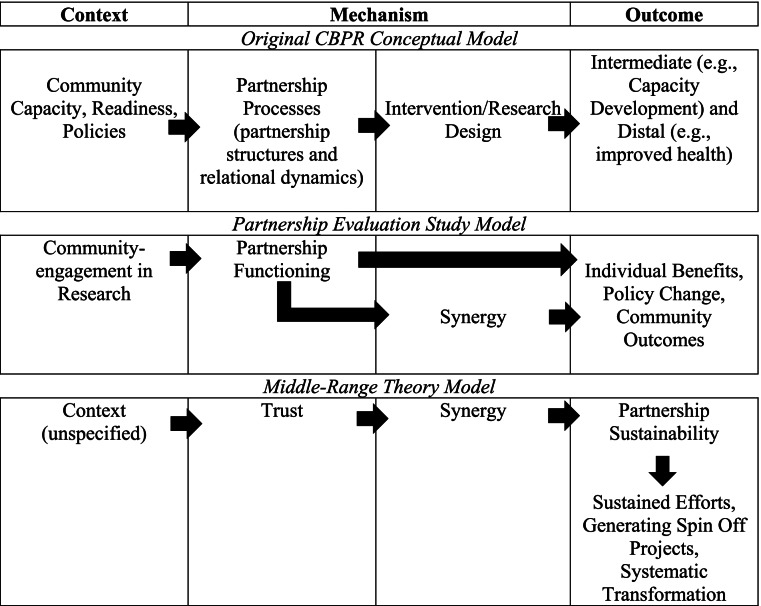
Paths in the Context, Mechanism, Outcomes of Three Prior CBPR Models

**Fig. 1:**
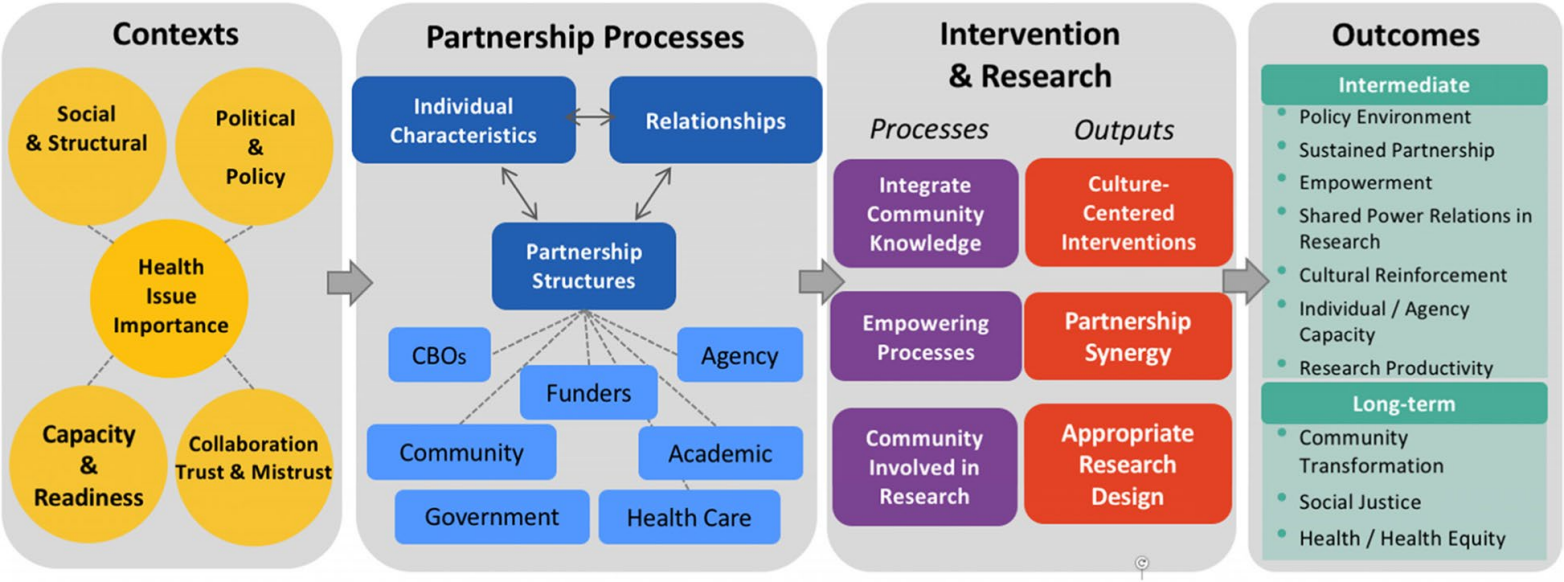
CBPR Conceptual Model

Building on an earlier model [12], extensive literature reviews, a survey of CBPR practitioners, and consultation with a national advisory board of community and academic CBPR experts, we created a CBPR Conceptual Model [2, 17]. This theory-informed model includes four domains: context, partnership processes, intervention/research, and outcomes (system/capacity and health outcomes). Context includes university/community capacity, readiness for change, socio-economic factors, historical collaboration, and policies which frame the partnership processes. Partnership processes include individual partner characteristics, partnership structures, and relational dynamics. Partnering processes influence the action domain of intervention/research which reflects empowering processes, degree of community engagement in research, and integration of community knowledge. Intervention/research contributes to intermediate outcomes (e.g., capacity development and change in power relations) and distal or long-term outcomes (e.g., improved health and health equity) for the individual partners and the community. The model has undergone community consultations [18] and testing using a U.S.-based study of 200 CBPR projects [19] followed by responsive revisions through pragmatic use to guide planning and evaluation [20]. The CBPR Conceptual Model embeds the importance of pathways throughout, with contexts influencing the mechanisms chosen (i.e., with whom and how partnering processes are implemented, combined with action taken in intervention/research design) leading to outcomes (see Fig. 1 with a more comprehensive model available from http://engageforequity.com).

The Partnership Evaluation Study of federally-funded mental health research centers [11, 15] was among the first to use rigorous empirical research to examine CBPR processes and outcomes, particularly examining the impact of community engagement on outcomes. The study logic model emphasized two key mechanisms—synergy and community engagement in research. Synergy results from partnerships functioning to accomplish more collectively than what individual members could achieve independently. Community engagement in research is the degree of community partnership involvement in the various research tasks of the project. The study found that community engagement in research influenced partnership functioning, which affected synergy. Further, community engagement in research and synergy had direct positive impacts on outcomes including individual partner benefits, policy changes, and community outcomes [11].

A third realist evaluation model, expressed as a middle-range theory of pathways between contexts, processes, and outcomes, was derived from literature and interviews with CBPR investigators [10, 16]. This research specifically examined how trust and synergy facilitate health improvement and community transformations. They found that trust among partners led to synergy. Synergy then had impacts on partnership sustainability, which then led to sustaining efforts to improve community health, generating spin off projects, and systematic transformation.

The most recent iteration of the CBPR Conceptual Model integrates the mechanisms identified in these previous models while focusing on two other key elements [2]. The addition of these two elements, and development from a previous structural equation model [19], results in a hypothesized model including eight latent constructs. There are two contextual constructs. Contextual Capacity includes the skills and resources of a partnership [21]. This construct builds on our earlier work by including community history of organizing and bridging differences between community and academic partners. Structural Governance is one of the two new elements and refers to the means by which resources are shared and decisions are made for the benefit of the community [22, 23]. Structural Governance extends our previous work by combining three distinct variables into one single construct: the organization or entity that approves the project on behalf of the community, engages in joint decision-making on project resources, and shared project budget.

Partnership processes now has two key constructs, one of which is our second new element. Commitment to Collective Empowerment is grounded in CBPR’s foundation of Freirean reflection/action cycles and literature on community empowerment [24, 25]. It reflects partner commitment to CBPR principles, the fit to community, people’s influence, and their critical reflection to leverage community resilience to improve health outcomes [2, 26]. Commitment to Collective Empowerment replaces partnership structural values from the earlier structural equation model. Relationships, within partnership processes, references the group dynamics within the partnership including such elements as leadership, dialogue, conflict resolution, and trust [21, 27]. This construct is consistent with the previous structural equation model.

Intervention/research and outcomes also both include two constructs. Synergy is conceptualized and operationalized in a manner consistent with the Partnership Evaluation Study and the earlier structural equation model [11, 21]. Community Engagement in Research Actions (CERA) builds off of the Partnership Evaluation Study and our earlier model by adding community action along with other research components [11, 21]. Partnership and Partnership Transformation are intermediate outcomes of benefits to individuals and agencies, change in power relations, and partnership sustainability [21]. Projected Outcomes are long-term or distal changes such as policy change, social transformation, and health improvement [21]. These outcomes are consistent with the previous structural equation model with the constructs simply being renamed.

**Fig. 2:**
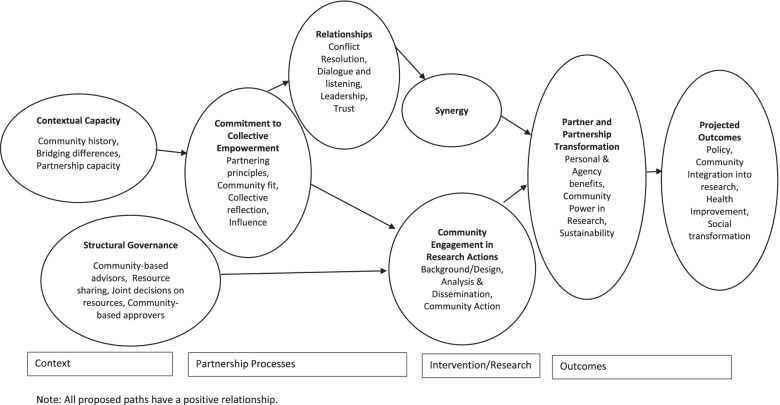
Hypothesized Model

The hypothesized associations among the constructs is displayed in Fig. 2. Briefly, Contextual Capacity is associated with Commitment to Collective Empowerment, which leads to Relationships and then Synergy. Structural Governance and Commitment to Collective Empowerment have paths to CERA. Both CERA and Synergy have paths to Partner and Partnership Transformation, which then leads to Projected Outcomes. All of these hypothesized paths are positive relationships.

The purpose of this study was to test specific theoretical mechanisms within the CBPR Conceptual Model and to identify the key contributors to these mechanisms. The first study aim was to test a structural equation model derived from the CBPR Conceptual Model to identify whether the paths among the domains are consistent with the theoretical mechanisms. Building on the structural equation model results, the second aim was to examine whether context and process constructs, independently or collectively, aligned with the key intervention/research constructs of partnership synergy and CERA. Thus, this study aims to identify both overall pathways and critical drivers of how partnerships can best work together to produce their desired health outcomes and societal impact.

## Methods

We used data from a U.S-based sampling frame of 413 CBPR or CEnR projects as part of the Engage for Equity project [21, 24, 28]. The sample was drawn from the National Institutes of Health (NIH) RePORTER database of federally-funded projects in 2015 along with Centers for Disease Control and Prevention (CDC)-funded Prevention Research Centers, the Native American Research Centers for Health projects funded by multiple NIH Institutes, funded projects from the Patient-Centered Outcomes Research Institute, and funded projects from three training institutes. Principal investigators and project team members from selected projects completed a two-stage cross-sectional online survey. Ethics approval was provided by the University of New Mexico’s Institutional Review Board (#16–098).

### Research design and sampling

The research design involved multiple stages that are detailed elsewhere with brief review provided here [21, 23, 28]. Stage one involved selecting the projects from the databases if they included a research grant mechanism (e.g., R or U mechanisms) and had at least two years of funding remaining. Two years of funding was necessary as a later stage of the Engage for Equity grant invited some projects to a workshop for enhancing collective reflection and partnership processes [24]. This resulted in a sample of 377 community-academic partnership projects. In addition, 36 new partnerships from three training institutes were included. Stage two included an internet-based key informant survey sent to principal investigators or designates of all identified projects in 2016–17 for the initial sample and in 2018 for newly developed partnerships; 53% of the principal investigators from the original sample responded with 179 completed surveys, and 86% (*n* = 31) of new partnerships responded and completed the survey. The respondents were asked to identify up to six partners (i.e., project team members; four community and two academic) to participate in the community engagement survey.

Third, the community engagement survey was sent to 611 participants in 2016–17; 381 (62%) consented and completed at least 75% of the survey. A total of 133 invitations were sent in 2018 to the new partnerships, and 76 (57%) consented and completed at least 75% of the survey. Overall, there were 186 community partners and 266 academic partners (with 1 not identifying). The projects predominantly worked with communities of color including Black/African-American (57%), Hispanic/Latino (45%), American Indian/Alaska Native (31%), and Asian (18%) (numbers exceed 100% because some projects involved multiple populations). We provided the surveys in English and Spanish to participants to be inclusive of the large number of potential participants working in Spanish-speaking communities. We used a translation/back translation procedure to ensure equivalence. Three community partners completed the survey in Spanish.

### Measures

Measures for this project [21] were based on our prior work [22, 29] and included two scales from each of the four domains of the CBPR Conceptual Model (with the majority having subscales as listed in Fig. 2). Context scales were Contextual Capacity and Structural Governance. Partnership process scales were Commitment to Collective Empowerment and Relationships. Intervention/research scales were CERA and Synergy, and outcome scales were Partner and Partnership Transformation (intermediate) and Projected Outcomes (long term). Descriptive statistics and psychometric validation of these scales are found elsewhere [21, 23] with the standardized coefficients and standard errors presented in Table 2. Aside from Synergy which had no subscales, each of the remaining scales was comprised of three to four subscales. Subscale scores and the scale score for Synergy were calculated as the mean across non-missing items, and, aside from Structural Governance, response options for items were all on a six-point unipolar rating scale (e.g., “not at all” to “to a complete extent”) or on a seven-point bipolar rating scale (e.g., “completely disagree” to “completely agree”).

**Table 2:** Measurement and Structural Equation Model

	Coef^a^	SE^b^
**STRUCTURAL MODEL**		
Contextual Capacity - > Commitment to Collective Empowerment	0.72	0.04
Commitment to Collective Empowerment - > Relationships	0.84	0.03
Relationships - > Synergy	0.28	0.07
Commitment to Collective Empowerment - > Synergy	0.63	0.07
Synergy - > Partner and Partnership Transformation	0.55	0.05
Structural Governance - > CERA	0.19	0.05
Commitment to Collective Empowerment - > CERA	0.61	0.04
CERA - > Partner and Partnership Transformation	0.40	0.06
Partner and Partnership Transformation - > Projected Outcomes	0.88	0.03
**MEASUREMENT MODEL**		
Contextual Capacity		
Bridging differences	0.78	0.03
Community history	0.64	0.04
Partnership capacity	0.82	0.03
Commitment to Collective Empowerment		
Partnering principles	0.95	0.01
Community fit	0.82	0.02
Influence in the partnership (Voice)	0.47	0.05
Collective Reflection	0.63	0.03
Relationships		
Leadership	0.88	0.02
Dialogue and listening	0.68	0.04
Conflict resolution	0.72	0.04
Trust	0.75	0.03
CERA		
Background & design	0.86	0.02
Analysis & dissemination	0.92	0.01
Community action	0.84	0.02
Partner and Partnership Transformation		
Personal benefits	0.61	0.05
Agency benefits	0.73	0.03
Community power in research	0.66	0.04
Sustainability	0.60	0.04
Projected outcomes		
Policy	0.91	0.01
Community integration into research	0.77	0.03
Social transformation	0.83	0.02
Health improvement	0.73	0.03
Error covariances		
Partnering principles, Community fit	0.21	0.10
Conflict resolution, Participation	0.54	0.07
Personal benefits, Agency benefits	0.27	0.07

^a^ Standardized regression coefficients are all *p* < .001 except *p* = .03 for error covariance between partnership principles and community fit; ^b^ Standard Error adjusted for project-level clustering of participants

### Data analysis

The final analysis was based on 453 partners (379 in first and 74 in second samples) in 165 projects (139 first and 26 s). The sample size for analysis includes all projects that had a completed key informant survey (with a Structural Governance score) and at least one community engagement survey completed. All statistical analyses were conducted in Stata 16.

In order to address the first aim, a planned structural equation model, based on our prior work in this area [19], was developed and assessed in multiple steps. Structural equation modeling is a common approach to use when testing the paths of a conceptual model such as in the current study [30, 31]. An initial model was fit via maximum likelihood with missing values estimation that included the factor score for Structural Governance and the scale score for Synergy as manifest variables and all other scales as latent constructs, manifesting in their respective subscale scores. Error covariance terms were included in this model only between subscales that had error covariance from the psychometric testing [21]. Modification indices, along with conceptual review, were used to identify at most one additional path or error covariance term to add to this model. Thresholds for “good” fit in the finalized model were RMSEA< .06, and CFI, TLI > .95, and thresholds for “acceptable” fit were RMSEA<.08 and CFI,TLI > .90 [31]. Model sensitivity to missing-ness was assessed by examining consistency of model fit and coefficients to maximum likelihood estimation with listwise deletion. To account for the nesting of partners within projects, cluster-robust standard errors were reported and incorporated into inferential tests for the finalized model.

In order to meet the second aim, fuzzy-set qualitative comparative analysis (FsQCA) models were assessed using Stata’s “fuzzy” package [32]. FsQCA is increasingly used in research related to identifying key factors for community engagement and health outcomes [33, 34]. FsQCA involves identifying a set of attributes which individually or in some configuration explain the extent to which a condition of interest is present using a case-oriented approach [35]. Due to a substantive interest in CEnR projects as cases, data for this aim was aggregated to the project level prior to analysis by calculating mean responses from partners within projects. In this study of 165 projects as cases, FsQCA involves examining various combinations of the four context and process scales to determine which best predict CERA and Synergy.

As raw scores, all manifest variables involved in the analysis were linearly re-scaled to range from 1 (indicating complete absence) to 6 (indicating complete presence). Scale scores, where applicable, were calculated as the mean across non-missing subscale scores; all scores were, then, “calibrated” (0 = fully absent to 1 = fully present) using a standard logistic transformation [36], and means and standard deviations were used to describe both calibrated and uncalibrated scores. Alignment of the presence of attributional drivers of interest with the presence of an outcome was then determined by examining consistency and coverage scores. In FsQCA, “consistency” refers to degree to which some configuration of attributes is present in equivalent or *lesser* amounts than some condition, and, inversely, “coverage” refers to the degree to which some configuration of attributes is present in equivalent or *greater* amounts than some condition (i.e., consistency is lower when the attribute overpredicts the outcome and coverage is lower when the attribute underpredicts the outcome). As is common FsQCA practice, .80 was used as a threshold for “strong” consistency and coverage. It is important to consider a balance of both consistency and coverage as often high scores in one results in low scores in the other. We focused on consistency and coverage of the context and process scales with both Synergy and CERA as these were key mediators in the structural equation model between context/process and outcomes.

## Results

### Structural equation model

**Fig. 3:**
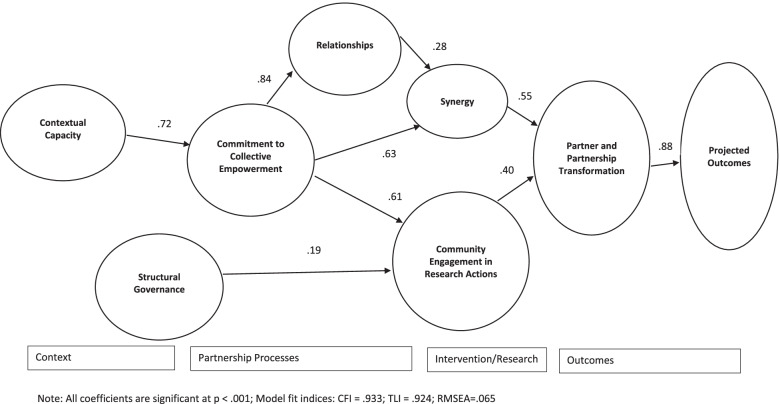
Structural Equation Model

Table 2 includes the standardized regression coefficients for each subscale within a scale in the model. Figure 3 illustrates the pathways included in the data-generated model and their magnitude, direction, and statistical significance. The initial model prior to modification achieved acceptable fit, CFI = .923, TLI = .913, and RMSEA = .070. However, a modification was strongly warranted for a path from Commitment to Collective Empowerment to Synergy resulting in an improved fit, CFI = .933, TLI = .924, and RMSEA = .065. Two predominant paths from context to outcomes were identified, and all associations were positive and statistically significant. First, Contextual Capacity was associated with Commitment to Collective Empowerment (.72), a focal driver in this pathway. Commitment to Collective Empowerment, in turn, was directly associated with CERA (.61) and was also associated with Synergy both directly (.63) and indirectly, via Relationships (.84 for Collective Empowerment to Relationships and then .28 to Synergy). Synergy (.55) and CERA (.40), in turn, both contribute to Partner and Partnership Transformation, which then is associated with Projected Outcomes (.88). Second, Structural Governance was associated with CERA (.19) which was associated with Partner and Partnership Transformation (.40) and then Projected Outcomes (.88).

### FsQCA

Relationships were, on average, the most present attribute having a mean calibrated score of .85 with Commitment to Collective Empowerment, Synergy, and Contextual Capacity all at or above .70. Further, projects had more absence than presence of CERA and Structural Governance with both conditions showing mean calibrated scores below .50 (See Table 3). All project context and process attributes were strongly consistent with Synergy. Further, with the noted exception of Structural Governance, the context and process attributes also had strong coverage of Synergy. In terms of overall alignment with Synergy, Commitment to Collective Empowerment had the closest balance of strong coverage and strong consistency and demonstrated, across projects, calibrated scores strongly equivalent to extent of project Synergy. The Supplemental File provides a technical appendix for the FsQCA.

**Table 3:** FsQCA. Descriptive statistics and alignment measures of context and process conditions and Synergy and CERA

	Raw score	Calibrated score	Synergy	CERA
Construct	*M* (*SD*)	*M* (*SD*)	(Consistency, Coverage)	(Consistency, Coverage)
Contextual Capacity	4.34 (0.74)	0.70 (0.17)	(0.95, 0.88)	(0.66, 0.97)
Structural Governance	3.11 (1.53)	0.42 (0.32)	(0.92, 0.51)	(0.77, 0.67)
Commitment to Collective Empowerment	4.75 (0.59)	0.80 (0.12)	(0.93, 0.97)	(0.60, 0.99)
Relationships	5.07 (0.55)	0.85 (0.10)	(0.89, 1.00)	(0.56, 0.99)
Synergy	4.61 (0.73)	0.76 (0.16)		
CERA	3.45 (1.02)	0.48 (0.24)		

Raw scores for constructs can range from 1 to 6 and calibrated scores can range from 0 to 1

In contrast, none of the individual context or process attributes demonstrated strong consistency with CERA. However, 13 of the 16 configurations of attributes (see Table 4) were strongly consistent with CERA and had consistency scores that were significantly greater than .80. Interestingly, 94% (155 of 165) of actual projects in this sample had configurations of context and process attributes that appeared in the bottom quartile of consistency with CERA. Only one of these lower quartile configurations, the presence across all four included attributes, had a consistency score that was significantly greater than .80; 36% (*n* = 59) of the sample had this configuration. This configuration also had the strongest coverage thus providing the best balance of consistency and coverage. The most common configuration (*n* = 81, 49%) in this sample (presence of strong Capacity, Empowerment, and Relationship practices and the absence of strong Governance practices), was the only configuration in this analysis showing a consistency score that was statistically lower than .80. These results suggest that the majority of projects had a configuration of contextual and process attributes that are incompatible with achieving a core principle of CBPR—to engage community members in research actions.

**Table 4:** FsQCA. Presence, consistency, and coverage of context and process conditions with CERA

Configuration	*n*	Consistency	Coverage
CGEr	0	0.97***	0.23
cGer	0	0.96***	0.22
cGEr	0	0.96***	0.23
CGer	1	0.96***	0.22
CGeR	0	0.94***	0.27
cGeR	0	0.93***	0.26
CgEr	1	0.92***	0.26
cgEr	0	0.91***	0.25
cger	0	0.91***	0.24
Cger	1	0.91***	0.25
cGER	6	0.89**	0.35
CgeR	1	0.86*	0.32
CGER	59	0.86**	0.66
cgeR	2	0.85	0.30
cgER	13	0.78	0.40
CgER	81	0.69***	0.66

*n* = number of partnerships with the configuration; Consistency is the degree to which the configuration of attributes is present in equivalent or *lesser* amounts than CERA, coverage is the degree to which the configuration of attributes is present in equivalent or *greater* amounts than CERA. C = Contextual Capacity, G = Structural Governance, E = Commitment to Collective Empowerment, R = Relationships. Capital letters for an attribute indicate presence of the attribute based on the calibrated score. Lower case letters indicate the complement, absence of the attribute in a project. *p < .05, **p < .01, ***p < .001

Further, the top quartile of most consistent configurations in Table 4, while showing complete variability in presence or absence of strong capacity and empowerment attributes, all included Gr—presence of strong Governance and absence of strong Relationship practices. This finding is congruent with Structural Governance showing the strongest consistency with CERA and Relationships showing the weakest consistency with CERA. Further, with the noted exception of Structural Governance, all project context and process attributes in Table 3 showed very strong coverage of CERA, effectively acting as upper bounds for the presence of CERA. Taken together, these FsQCA results reinforce the importance of Commitment to Collective Empowerment as a focal driver of Synergy and highlight the role of Structural Governance practices for the presence of CERA in CEnR and CBPR projects.

In summary, the purpose of this study was to test specific structural and relational theoretical mechanisms within the CBPR Conceptual Model in order to identify what pathways are most important for reaching health equity and other health outcomes. By combining two distinct analytic methods, the importance of both pathways and specific context and process conditions within the conceptual model were validated. The structural equation analysis of a robust data set including 165 CBPR projects and 453 individual partners, supported Commitment to Collective Empowerment, Synergy, and CERA as key mediating variables among context, processes, intervention/research, and outcomes. FsQCA identified Commitment to Collective Empowerment and Structural Governance as key conditions for Synergy and CERA respectively.

## Discussion

The findings of this study provide additional support for the growing research body around the CBPR Conceptual Model [2, 18, 22]. This study represents our second large-scale study to support the key constructs and premise of the model [24]. These two studies include nearly 400 CBPR or CEnR partnerships, providing a strong empirical foundation for the model itself and for the theoretical mechanisms and pathways within the model which have been established through prior research [10, 11, 15].

As researchers and practitioners increasingly utilize CEnR generally and CBPR specifically to work with communities to enhance health and health equity, this study also identifies two critical new drivers as promising practices for effective community and academic partnerships: Commitment to Collective Empowerment and Structural Governance. Commitment to Collective Empowerment was particularly important for Synergy with the FsQCA results showing that levels of collective empowerment essentially determine levels of synergy. Despite the attention paid to synergy in the literature [10, 11, 27], in our data set, Commitment to Collective Empowerment appears to be a more fundamental mechanism of CBPR than Synergy, reflecting informal processes created during partnership interactions. Commitment to Collective Empowerment consists of partnering principles, community fit, collective reflection, and influence [21].

Structural Governance was important for CERA in the FsQCA with all configurations including Structural Governance associated with high levels of CERA. This suggests the centrality of sufficient resources to enable community participation, which facilitates high integration of community knowledge [23]. In other words, Structural Governance helps to steward the project to ensure community members are engaged in the research and that there is a focus on community benefit [22]. Native communities have been leaders in this area with a strong focus on formal governance of CEnR projects through institutional review boards and formal approval processes during governmental meetings [37, 38].

What Structural Governance and Commitment to Collective Empowerment mean in practice can be exemplified by the NIH-funded intervention, the Family Listening Program, based on a 20-year partnership between the University of New Mexico and three tribal communities [39]. While the partnership started with tribal approval processes and shared budgets with tribal IRBs and leadership, partners engaged in collective empowerment practices to build trust and norms of community members as drivers of the culture-centered intervention (community fit) and as decision-makers on program design and implementation (influence). Ongoing cycles of Freirian reflection on each action taken led to community member engagement in all research steps (CERA), including a recommitment to community governance in community members being co-presenters and co-authors of their own data and learnings. These processes reflect core theoretical foundations of CBPR and that result in partners being committed to the mission and vision of the partnership, ensuring there is synergy and authentic-partnered community engagement [2, 9, 40, 41].

In contrast to much of the literature on CBPR, relative to empowerment and governance practices, Relationships did not arise as a key driver of either synergy or CERA in the FsQCA analysis. Much of the CBPR literature emphasizes high-quality partnership processes such as mutual learning and respect [10–12, 42]. Projects in this sample tended to report very high presence of positive Relationships, in excess, especially, of levels of CERA or Structural Governance. However, it is important to note that the FsQCA results also indicated that there were multiple configurations of context and process conditions that were highly consistent with both Synergy and CERA. Thus, there is not one ideal or perfect approach for CBPR.

### Limitations

First, with projects U.S. funded and implemented, generalizability of this study may be limited to the U.S. context. Similarly, the language of our study was almost exclusively in English so that may introduce some bias. The CBPR conceptual model has been translated into Spanish, Portuguese, German, and Swedish and applied to CBPR projects in other nations, with emerging studies about its use [43–45]. Second, the surveys were cross-sectional and thus direct causal relationships among the variables cannot be established. The relationships are consistent with the conceptual model, but longitudinal evidence is necessary to more strongly substantiate the theoretical mechanisms in the model.

Third, most of the measures in the community engagement survey are perceptual as it was difficult to obtain “objective” measures of the variables. The key informant survey did provide more “objective” measures about key facets of the project, particularly in the area of Structural Governance. Additionally, the participants are community and academic stakeholders who are deeply engaged with a strong sense of their own processes and impacts, yet they were also nominated by project leaders which introduces some bias in responses. Future research should identify actual outcomes over time to more strongly identify key context, process, and action intervention/research variables that link to intermediate and long-term outcomes. Future research can consider whether there are differences in the consistency and coverage of Structural Governance and Commitment to Collective Empowerment in different types of communities (e.g., Native versus non-Native partnerships), and if there are mediating factors between structural governance and CERA.

### Public health implications

CBPR and CEnR are important strategies to address health equity and structural racism. Yet, while the CBPR literature and practice tends to emphasize Relationships and Synergy, the current study demonstrates that they are necessary, but not sufficient factors for producing CBPR generated health outcomes. Structural Governance and Collective Empowerment were more foundational and impactful for outcomes than Relationships and Synergy. Structural Governance practices remain underutilized, with only 42% of projects identifying its presence, especially since communities (and funders) are increasingly demanding approval processes, shared budgets, and community decision-making and stewardship.

Other engagement models continue to be developed, with most recently a 2022 National Academy of Medicine model which emphasizes four outcome-oriented domains that contribute to equity through transformed systems for health [46]. However, our CBPR Conceptual Model and the analysis of metrics and measures based on its four domains of the CBPR Conceptual Model in this study and elsewhere [21, 24, 28] offers four major advances to the science of CEnR: a) the value of an engagement framework that enables the study of pathways; b) the importance of conditions or contexts that influence the choice of pathways and practices, with decisions possibly depending on the extent of Structural Governance, stage of partnership, or other local community organization strengths; c) the importance of both relational and structural pathways for partnership success in reaching outcomes; and d) the value of Commitment to Collective Empowerment as a condition to deep engagement of community partners. This commitment reinforces the value for all partners to engage in reflexivity and self-evaluation as an interpretative dynamic practice of their own strengths and weaknesses in order to enhance shared power within the partnership and towards their external equity goals. While these results came from an NIH study on research practices and outcomes, pragmatic application of the CBPR Conceptual Model, with its pathways and metrics, are being currently tested with community coalitions, health councils, and other community engagement projects with promising results as a planning, visioning, and evaluation tool. These practices are also being explored in a current PCORI engagement study examining institutional facilitators and barriers to equity-based community and patient-engaged research within academic health institutions. Future research could assess their impact, which may differ depending on stage of partnership, population, geography, setting, or the meaning partners ascribe to their own dynamic processes and outcomes.

While the study does not support a single “best practice” pathway model, these two promising practices should be considered for partnership building, planning, and assessment. CBPR partners can integrate collective reflection and meaning making on these processes as such reflection can strengthen partnerships. Health systems can integrate these practices into their patient engagement efforts. Funders, such as NIH, should seek evidence of these two practices in applications, particularly evidence that there is Structural Governance including shared resources with communities, to accomplish goals of health equity.

## Conclusions

The purpose of this study was to test specific theoretical mechanisms within the CBPR Conceptual Model and to identify the key contributors to these mechanisms. The use of different analystical methods facilitated recognition of complexity within the CBPR Conceptual Model. Avoiding a single analystical method rendered less likely the identification of a single pathway through which CBPR and CEnR partnerships confront public health challenges. The structural equation model and FsQCA illustrated that Commitment to Collective Empowerment and Structural Governance are key drivers in relational and structural pathways, respectively, among context, partnering processes/actions in intervention/research, and outcomes in community-academic partnerships. These findings support the theoretical grounding of the CBPR conceptual model, which also includes Synergy and CERA. While the results expand expectations beyond a “one size fits all” for reliably producing positive outcomes, there is strong support that Collective Empowerment and Structural Governance are important for partnership success in address health outcomes and health equity.

## Supplementary Information

**Additional file 1.**

## Data Availability

Individual participant data that underlie the results reported in this article, after de-identification (text, tables, figures, and appendices), will be made available by request to nwallerstein@salud.unm.edu; to gain access, data requestors will need to complete a data access agreement.
