# Supplementary material for: Exploring theoretical mechanisms of community-engaged research: a multilevel cross-sectional national study of structural and relational practices in community-academic partnerships
Source: Int J Equity Health. 2022 May 2;21:59. doi: 10.1186/s12939-022-01663-y (PMC9063068; doi:10.1186/s12939-022-01663-y)
Supplement: Supplementary file 1 — Additional file 1. [file 12939_2022_1663_MOESM1_ESM.docx]

**Technical Appendix**

**Fuzzy set qualitative comparative analysis**

Standard references on fuzzy set qualitative comparative analysis (FsQCA) tend to emphasize framing results in terms of necessity and sufficiency, language which can be misleadingly causal in the context of cross-sectional data analysis. In this technical appendix, we take an alternative, yet mathematically equivalent approach, using data-based figures and tables to review FsQCA concepts and presenting consistency (degree of sufficiency) and coverage (degree of necessity) firmly in terms of, respectively, lower and upper bounds.

*Calibration*

Calibration in FsQCA refers to a process of assigning attributes of cases to a zero to one scale, from complete absence to complete presence. See Figures A and B (below) for plots of calibrated scores across cases for Contextual Capacity, Commitment to Collective Empowerment, Structural Governance, Relationships, and Synergy.

*Consistency and Coverage*

Mathematically, consistency in FsQCA is defined by the formula $\frac{\sum\min(x_{i},y_{i})}{\sum x_{i}}$ and coverage in FsQCA is defined by the formula $\frac{\sum\min(x_{i},y_{i})}{\sum y_{i}}$. Accordingly, consistency and coverage can both range from a miminum of zero to a maximum of one; an attribute X has maximum *consistency* with a condition Y if and only if the calibrated scores for X always form a *lower* bound for the calibrated scores for Y; and an attribute X has maximum *coverage* with a condition Y if and only if the calibrated scores for X always form an *upper* bound for the calibrated scores for Y. Consistency scores decline from maximum toward zero to the extent to which values for X tend to exceed values for Y, and, conversely, coverage scores decline to the extent to which values for X tend to be lower than values for Y.

In Figure B, calibrated values for Relationships mostly slightly exceed calibrated values for Synergy, leading to an effective upper bound for Synergy, near perfect coverage (1.00), and lesser consistency (0.89). In contrast, calibrated values for Structural Goverance are usually quite a bit below calibrated values for Synergy, leading to an effective lower bound for Synergy, higher consistency (0.92), and lower coverage (0.51). In Figure A, calibrated values for Commitment to Collective Empowerment are, overall, quite strongly aligned with calibrated values for Synergy, having both high consistency (0.93) and high coverage (0.97). See Table 3 for further details.

Figures A and B also illustrate the ways in which an FsQCA approach differs from a more typical approach using linear regression to examine strength of relationships. A linear regression approach would be based on relative values and re-scaling and re-centering of scores would leave results unchanged. In contrast, FsQCA results are based on absolute calibrated values and are quite sensitive to data transformation. Also, consistency and coverage in FsQCA are based on one-sided bounds, quantifying the tendency to either be less than (consistency) or more than (coverage). Linear regression, in contrast, is based on overall two-sided nearness and does distinguish the directionality of nearness to fitted values.

*Complex configurations*

A strength of FsQCA is the ability to explore consistency and coverage of complex configurations with conditions of interest, allowing for the consideration of various interactions among attributes. These configurations are created through combinations of the logical operations of negation (x is 1-X, the complement of X), conjunction (XY is max(X,Y), the intersection, X and Y), and disjunction (X Y is min(X,Y), the union, X or Y). For instance, as in Table 4, with G = Structural Governance and R= Relationships, the configuration Gr denotes the amount of presence of strong Structural Governance *and* amount of absence of strong Relationships, while G r denotes presence of strong Structural Governance *or* absence of strong Relationships. Disjuction tends to increase consistency while reducing coverage; conversely, conjunction tends to decrease consistency while increasing coverage. For instance, looking, as in Table 4, at the presence of strong Community Engagement in Research Actions as the condition of interest, G on its own (see Table 3) has consistency of 0.77 and coverage of 0.67, Gr has consistency of 0.96 and coverage of 0.23, and G r has consistency of 0.72 and coverage of 0.76.


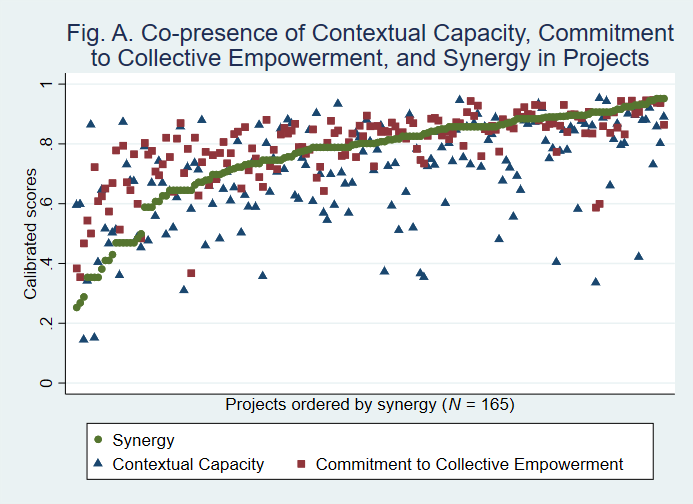


Figure A. Alignment between the presence of select context and process conditions and the presence of Synergy across 165 CEnR projects in the study sample, 2016-2018.


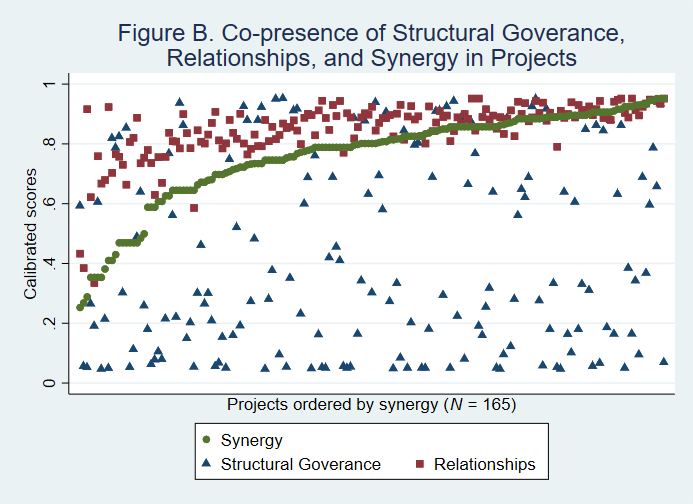


Figure B. Alignment between the presence of select context and process conditions and the presence of Synergy across 165 CEnR projects in the study sample, 2016-2018.
